# Cycling in Warsaw, Poland – Perceived enablers and barriers according to cyclists and non-cyclists

**DOI:** 10.1016/j.tra.2018.04.014

**Published:** 2018-07

**Authors:** Katarzyna Iwińska, Malgorzata Blicharska, Livia Pierotti, Marko Tainio, Audrey de Nazelle

**Affiliations:** a Collegium Civitas, pl. Defilad 1, PKIN, 00-901 Warsaw, Poland; b Natural Resources and Sustainable Development, Department of Earth Sciences, Uppsala University, Villavägen 16, 75 236 Uppsala, Sweden; c Centre for Environmental Policy, Imperial College London, 13 G7 Princes Gardens, London SW7 1NA, UK; d UKCRC Centre for Diet and Activity Research (CEDAR), MRC Epidemiology Unit, University of Cambridge School of Clinical Medicine, Cambridge, UK; e Systems Research Institute, Polish Academy of Sciences, ul. Newelska 6, 01-447 Warsaw, Poland

**Keywords:** Active transport, Barriers to cycling, Urban cycling, Cyclist perception, Infrastructural changes in the city

## Abstract

Cycling in urban environments provides many benefits to people. However, planning of cycling infrastructures in large cities faces numerous challenges and requires better understanding of both the factors enabling cycling as well as barriers to it, determined by particular local context. While there is a growing body of research that tackle the bike transport related questions in Western Europe and the USA, there is relatively little research on that in Central Eastern Europe (CEE), in post-communist countries. In this study we used qualitative and quantitative methods to explore urban cyclists and non-cyclists opinions about the cycling, the perceived problems and obstacles, and perception of the on-going changes in bicycle transportation system in Warsaw, Poland. Although many people see potential advantages of cycling, it is mostly perceived as a leisure time activity. Those who do utilitarian cycling are more acutely aware of the benefits, such as rapidity and flexibility of this mean of transport. The main perceived barriers are linked to lack of good cycling infrastructure in the city, the feeling of insecurity linked to the behaviour of drivers, and to maintenance during winter. In conclusion, our research highlights both the opportunities and challenges linked to the development of improved cycle transportation system, suggesting the need for a range of policies, from the infrastructure improvements and comprehensive planning of the whole transportation system, to improving the driving culture that would support feeling of security of the cyclists.

## Highlights

• Most of the cycling in Warsaw is recreational.
• Lack of proper infrastructure and safety on roads prevent more cycling.
• Weather is a more important barrier for recreational than utilitarian cyclists.
• A comprehensive set of policies is needed to make Warsaw “bike-friendly”.

## 1 Introduction

Cycling in urban environment can provide many benefits such as preventing sedentary lifestyle and associated health problems (Cervero and Duncan, 2003, Racioppi et al., 2013, Goetschi et al., 2016). There is a large body of observational and modelling research indicating that moderate daily physical activity, such as cycling, provides numerous health benefits (e.g. Andersen et al., 2000, Rojas-Rueda et al., 2011, Wen et al., 2011, Woodcock et al., 2011). Moreover, cycling is perceived as a cost-effective way to exercise (Sevick et al., 2000) and to travel (Gossling and Choi, 2015). It is also a more environmentally-friendly transport mode as an alternative to oil-related transportation (Burke and Bonham, 2010), helping to reduce urban air pollution (Di Lonardo et al., 2013, de Nazelle et al., 2011). Planning for sustainable cities that support various modes of transport, including cycling, has become increasingly important particularly in the face of numerous environmental and social problems people face nowadays (Curtis, 2008, Rojas-Rueda et al., 2011). However, planning for cycling infrastructure in large cities faces numerous challenges and cycling is still a much underutilised means of transport in most cities (Pucher et al., 1999, Pucher et al., 2010, Moudon et al., 2005, Handy et al., 2014). Successful plans to improve health and sustainability through increased active transportation require a good understanding of characteristics such as: who cycles, where and why, and what are perceived factors enabling cycling as well as barriers to it.

There is a growing body of research on understanding determinants of cycling for transport. Most prominent features of successful cycling environments are related to provision of safe, convenient and connected cycling infrastructure. Indeed, effective strategies combine policies from restricting car use; providing well-connected, safe and dense bike lane networks; providing showers at work; and offering individualized marketing or targeted behaviour change campaigns to promote cycling (Winters et al., 2017). This need for multi-level interventions from individual to societal targets is supported by the literature showing how perceived environments can be more important than objectively derived environmental characteristics in explaining the choice to cycle (Braun et al., 2016).

Most existing research on determinants of cycling, however, is derived from Western Europe, Australia, and the USA (e.g. Cervero and Duncan, 2003, Rietveld and Daniel, 2004, Pucher et al., 2010, Margués et al., 2015), and there is relatively little research on cycling behaviour in the urban environments of post-communist countries in Central-Eastern Europe (CEE). These countries face particularly acute environmental problems linked to fast economic development, such as congestion and environmental problems (Klarer and Moldan, 1997, Blicharska et al., 2011). For example, according to the TomTom Congestion Index (2017)^1^ Warsaw is on the tenth most congested city in Europe with average daily congestion level of 37% (reaching 65% in the mornings and 72% in the evenings). Since population and employment in Warsaw is growing, congestion is expected to become more common in the future. Increased use of active transport could be one solution to mitigating that problem. However, although Warsaw has seen rapid change in cycling in the last decade, there are still relatively few people cycling in the city, especially compared to Western European cities. This is similar to other CEE countries, where relatively low share of people cycle in urban areas (see e.g. Puhe and Schippl (2014), comparing Budapest, Copenhagen and Karlsruhe). The comparison to some CEE cities with available data from EPOMM Modal Split Tool indicates that Warsaw is representative for other large CEE cities with over one million inhabitants (see Table S1 in Appendix).

In this study we investigate the bicycle use in the city of Warsaw, Poland. Using both quantitative and qualitative research methods, we investigate the cycling habits of the different types of urban cyclists, their opinion about the advantages of cycling in the city and the perceived problems and barriers to cycling. Additionally, we look into differences in perception between cyclists and non-cyclists in the city. Our aim is to explore characteristics and perceptions towards cycling of different types of cyclists and non-cyclists in Warsaw, in view of assessing how to best address cycle promotion in an urban Eastern European setting.

## 2 Methodology

### 2.1 Description of the study area

The city of Warsaw covers an area of 517 square kilometres with a population of 1.7 million people (GUS, 2016). The climate is a humid continental climate with cold winters and warm summers. The landscape is relatively flat with the highest point being 122 m above sea level and an average elevation of 100 m above sea level (Polish Geological Institute, 2009). Flat and mild climate indicates that Warsaw provides good environmental conditions for cycling.

According to “The Warsaw Traffic Study in 2015” (which is a summary of municipality transportation research in Warsaw) the vast majority of trips performed by Warsaw residents are those of internal nature (96%) (WBR, 2015). The overall mobility rate of Warsaw residents is 2 trips per day, which is nearly 3.4 million trips (3 348 336) every day. In 2015 almost one in five (18%) of all journeys were made on foot, 32% by car, 47% by public transportation, and 3.1% of travels are performed by bicycle (WBR, 2015).

Cycling in Warsaw has increased from 0.9 to 3.8% in the past ten years (WBR, 2005–2015). The length of bicycling paths and lanes increased from 275 to 361 km between 2010 and 2013, to now 457 km in 2017 (leading to a bike network density of 0.27 km for 1000 Warsaw citizens)^2^ , with another 74 km planned to be built by 2021 (Warsaw Bike Routes Development Program, WRR, 2015). The number of bicycle parking facilities increased during the same time from 117 to 267.

### 2.2 Study methods

Our approach to investigating cycling in Warsaw was to combine information from both quantitative data (surveys) and qualitative (individual and focus group interviews), as our research questions concerned not only the amount of different types of cyclist but also their perception on risk, potential enablers and barriers of cycling. Thus, the results lead to a broad spectrum of knowledge about infrastructural and cultural factors influencing travel behaviour.

#### 2.2.1 Quantitative surveys

Two surveys collecting data on cycle use and on perceptions and attitude towards cycle travel in the city were conducted between December 2012 and January 2013 (Table 1). First, we conducted a door-to-door pen and paper questionnaire based on a random sample of 600 inhabitants of Warsaw, representative of the population aged between 18 and 75 (henceforth referred to as the “random sample survey”). We made use of an address database to choose the respondents, and used age and gender as criteria for representativeness. The questionnaire took ca. 15 min to complete. Second, we conducted an on-line computer assisted web interview survey based on a purposive sample of 579 citizens of Warsaw who had indicated using the bike for recreational or transportation purposes at least once within the past 6 months in a preceding population survey of 15–65 year olds, conducted as part of the nationwide research panel “Ariadna” (http://panelariadna.pl/). We will henceforth refer to this second survey as the “cyclist survey”. Of the 579 respondents of the cyclist survey, 74 responded in our survey that they had not participated in any cycling activity in the previous 6 months and were therefore excluded from the remainder of the analysis, leaving 505 responses for the analysis.

**Table 1:** Characteristic of the two samples (random and cyclist) of participants. Data are presented as median unless otherwise stated. VPA = Vigorous Physical Activity; MPA = Medium Physical Activity; SD = Standard deviation; IQR (interquartile range).

Characteristic	Random sample (N = 561)				Cyclist sample (N = 505)			
	Total	Non-cyclist n = 258	Cyclist n = 303	p-value	Total	Recreational n = 207	Utilitarian n = 298	p-value
Female, n (%)	303 (54%)	145 (56%)	158 (52%)		214 (42%)	107 (52%)	107 (36%)	
Male, n (%)	258 (46%)	113 (44%)	145 (48%)	P = 0.381	291 (58%)	100 (48%)	191 (64%)	P < 0.001
Age mean (SD)	48 (15.2)	54 (15.8)	44 (13.1)	P < 0.001	31 (10.3)	32 (10.6)	31 (10.1)	P = 0.135
Total duration cycling (minutes per week)	NA	NA	NA	NA	23 (28)	16(18)	28(32)	P < 0.001
Total duration (minutes per week) of VPA	80 (280)	90 (180)	65 (280)	P = 0.527	180(280)	165(180)	180(280)	P = 0.433
Total duration (minutes per week) of MPA	100 (300)	72 (313)	120 (298)	P < 0.001	120(300)	100(313)	140(298)	P = 0.126
Walking at least 10 min, n daysMean (SD)	3.5 (2.7)	2.9 (2.6)	4.1 (2.7)	P < 0.001	4.72 (2.2)	4.68 (2.2)	4.75 (2.2)	P = 0.721
BMI mean (SD)	24.4 (3.8)	25.1 (4.0)	23.9 (3.5)	P < 0.001	24.4 (4.4)	24.3 (4.5)	24.5 (4.3)	P = 0.588
BMI categories, n (%)				P = 0.042				P = 0.011
Underweight, n (%)	13(3%)	5 (2%)	8 (3%)		28 (6%)	17 (8%)	11 (4%)	
Normal weight, n (%)	284 (57%)	110 (51%)	174 (61%)		276 (55%)	98 (48%)	178 (60%)	
Overweight, n (%)	199 (40%)	100 (47%)	99 (35%)		197 (39%)	88 (43%)	109 (37%)	

Both surveys had a similar structure and shared some common questions with regards to socio-demographics (age, sex, education and income, weight, height), travel habits (duration and purpose of travel by bicycle in past 7 days), physical activity levels, and perceptions with regard to cycling-related social and physical environment. The questions were derived from the Transport Air Pollution and Physical ActivitieS (TAPAS) project travel survey (Braun et al., 2016, Cole-Hunter et al., 2015).

Prior to analysing the survey data, data exploration was conducted for each of the variables (Appendix Table S2) to look for extreme values, errors and missing values by tabulating and summarizing each variable. Graphical plots were examined, and any extreme values were removed.

In the random sample, respondents were categorized as either non-cyclist or cyclist (who have cycled at least once in the last 6 months), while as in the cyclist sample, cyclists were categorized as either recreational (only biked for leisure in previous 7 days) or utilitarian (biked with an aim to go to specific places in previous 7 days) cyclist. Note that utilitarian cyclists may also have taken recreational bike rides (Appendix Table S2). Separately for both samples the crude association of socio-demographics, travel habits and perceptions of cycling were compared across these respondent categories using the χ^2^ test or Fisher’s exact test, means using one-way ANOVA and medians using the Kruskal Wallis Test. Finally, multivariable logistic regression models were applied to model the relationship between respondent category and each of the fifteen travel habits and perceptions variables individually after a priori adjustment for confounder. Age and sex were adjusted for a priori as they are common confounders; additionally, BMI was tested as a possible confounder by looking at the main exposure differences in the odds ratios.

#### 2.2.2 Qualitative study

To gain a more comprehensive picture of the cycling in Warsaw and to verify the findings from the surveys, we gathered qualitative data; we conducted a total of nine in-depth interviews (Kvale, 1996) with cyclists (4), transportation activists (4) and a scientific expert on transportation systems in Warsaw (1), and one focus group interview with 10 cyclists. In addition, we organised a workshop/group discussion with a panel of experts in bicycle transportation in Warsaw, representing both the city council and academics (from Warsaw municipality and Warsaw University of Technology). During the interviews and the workshop, we asked several open questions and used semi-structured interviews so that the interaction resembled a regular conversation (Flick, 2010). Semi-structured interviews were based on an interview guide which included questions concerning incentives for active transportation, perceived barriers, advantages and disadvantages of Warsaw public transportation system, infrastructural changes, etc. We used mixed qualitative and quantitative methods as the source triangulation, which allows for obtaining the data from different groups of people involved with the studied phenomenon in such a way as to grasp the differing perspectives surrounding the study subject (Patton, 1999). Using qualitative interviews in addition to quantitative surveys contributed to reducing potential biases on perspectives of real barriers to cycling and needs for improvements (no hypothesis or answers were given beforehand). The qualitative data were analysed using thematic analysis (Bryman, 2012) and were not subject to statistical analysis. When analysing results from the interviews, focus groups and the workshop, we used Atlas.ti – a qualitative data analysis software – to organize, classify, relate, and analyse all direct information from participants (quotes).

Next, we present factually results from the quantitative survey, followed by results from the qualitative study, which we then discuss jointly and in-depth in Section 4.

## 3 Results

### 3.1 Survey results

**Fig. 1:**
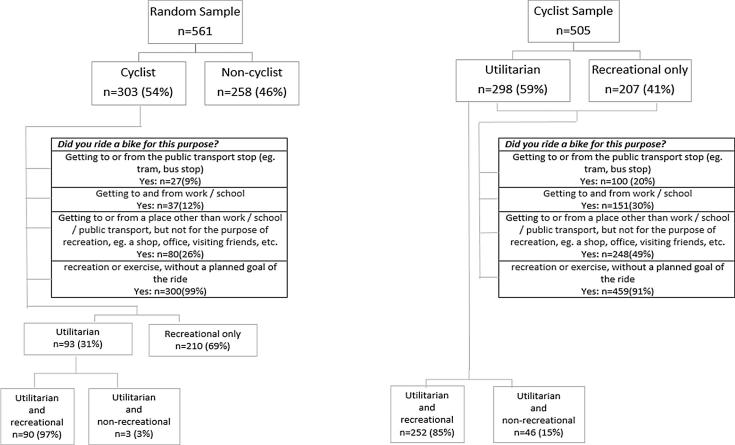
Cycling purposes in past 6 months.

According to the random survey, 54% of Warsaw residents cycled at least occasionally (i.e. at least once in the past 6 months). Cycling was most common as a recreational activity – most respondents who cycled used bikes only for recreational purposes (69%), and most (97%) of the cyclist who used bikes for utilitarian purposes also cycled for recreational purposes (Fig. 1). Also, in the cyclist sample recreational cycling was most common. While less than half of cyclists (41%) cycled only for recreation, most (85%) of the utilitarian cyclists also cycled for recreation (Fig. 1). The amount of cycling in the previous week was overall relatively low, and slightly higher for utilitarian (28 min) than recreational (16 min) cyclists. Note that only 9% (n = 43) of the cyclists sample reported any cycling in the past 7 days.

While the gender balance was similar between groups, cyclists were shown to be significantly (p = 0.001) younger than non-cyclist (on average 44 and 54 years old, respectively) (Table 1). Cyclists generally participated in more moderate-level physical activity (PA) and also walked significantly more than non-cyclists, with no statistically significant differences in vigorous PA duration. There was a statistically significant difference in BMI between cyclists and non-cyclists; 47% of non-cyclists were overweight compared to 35% of cyclists.

In comparison to respondents from the random sample identified as cyclists, respondents from the cyclist sample were on average younger (44 vs. 31 years old), more often male (48% vs. 58%), and performed more vigorous PA (65 vs. 180 min per week). However, both groups performed the same amount of moderate PA (120 min per week).

Analysis of the cyclist sample showed that utilitarian cyclists were significantly more likely to be male and have a normal weight compared to recreational cyclists, while both groups had similar average age. In general, utilitarian cyclists cycled more than recreational cyclists, but overall levels of PA were not statistically different (Table 1).

**Fig. 2:**
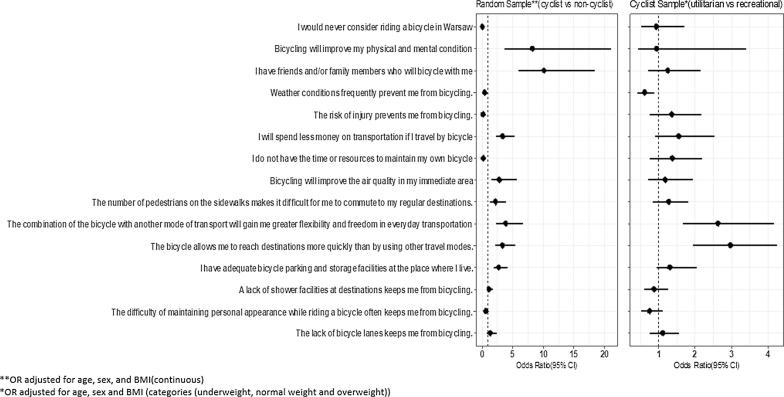
Odds ratio for agreeing with perception statements regarding physical and social aspects of cycling and 95% CI for utilitarian vs recreational cyclists in the cyclist sample (figure on the right) and cyclists vs non-cyclist in the random sample (figure on the left). Multivariable logistic regressions are adjusted for age, sex and BMI.

Multivariable logistical regression models assessed odds of agreeing with a series of perception statements regarding physical and social aspects of cycling for cyclists vs non-cyclists (random sample) and recreational vs utilitarian cyclists (cyclist sample) (see Fig. 2, Table 1, and Appendix Table S2). As controlling for BMI after adjusting for sex and age changed the estimated odds ratio for most of the models for both samples, BMI was included as a confounder (along with age and sex). When comparing recreational versus utilitarian cyclists in the cyclist sample, BMI was shown to differ significantly as a categorical variable (p = 0.011) but not as a continuous variable (p = 0.588). In the random sample BMI was shown to be significantly different between cyclists and non-cyclists when using a categorical variable (p = 0.042). The differences were even more strongly significant with BMI as a continuous variable (p < 0.001) (Table 1). Hence in multivariable analyses BMI was adjusted for as categorical variable in the cyclist sample, and as a continuous variable in the random sample. Either way, inclusion of the BMI did not change the significance, or the direction of the significant variables, of any of the perception questions, except for weather conditions becoming a significant deterrent for cycling vs non-cycling in the random sample (Appendix Tables S4 and S5).

Statistically significant differences were found for agreements with most perception questions when comparing cyclists to non-cyclists, from measures of the social environment to perceived benefits and barriers of cycling (see Fig. 2 for adjusted odds ratios and Appendix Table S2 for crude association descriptions). The two groups equally agreed *only* on the lack of adequate bike lanes, lack of showers at destinations and difficulties of maintaining appearance being barriers to cycling (around 85% and 62% of the sampled groups, respectively). Perceived supportive social environment (cyclist friends and family), higher convenience (faster and more flexible means to reach destinations), and benefits (physical and mental health benefits, air quality benefits, lower costs), as well as perceived lower risks (from traffic injuries), and barriers (weather, parking facilities, bicycle maintenance) were all associated with greater odds of being a cyclist. As noted above, the perceived weather barrier, high in both groups (over 75% of respondents agreed that this was a problem), lost its significance in comparing both groups when BMI was removed as a confounder. The greatest differences between cyclists and non-cyclists were found in their perceptions of social support and personal health benefits as motivators, and of risk of injury and bike maintenance as barriers for cycling (Appendix Table S3).

As expected, differences between recreational and utilitarian cyclists were much less pronounced than between cyclists and non-cyclists. Only weather condition was a significantly more important barrier for recreational than for utilitarian cyclists, and the possibility to reach destinations faster when cycling was significantly more important as a motivator for utilitarian cyclists (Fig. 2 and Appendix Table S3).

### 3.2 Qualitative interview results

Analyses of qualitative interviews of cyclists, activists and experts lead to identification of two groups of cyclists in Warsaw: (i) “occasional cyclists” and (ii) “hard core cyclists”. The first group consisted of people who treated cycling as an activity with both practical (e.g. commuting) and wellbeing benefits, being a part of a healthy lifestyle, cycling part-time in Warsaw but without “an activist approach” to cycling. For “hard core” users, cycling was a defining characteristic of their life style and ideology; they cycled throughout the whole year, whenever possible, either for professional purposes (i.e. delivery) or with utilitarian motives. The “hard-core” cyclists were involved in non-governmental organization (NGO) activities concerning cycling. As one of the respondents framed it: “This is also an issue like when for example I try to convince someone to ride a bicycle, I bring up the issue of social responsibility that we have towards our [shared] urban space. If everyone switched, or if a big group of people switched to bicycles, then the city would change right away. It would be a more pleasant city”.^3^ It is worth mentioning that road maintenance during the cold season (November–March) does not support cycling so that even “hard core” users sometimes suspend cycling (and some cycle on the road among other vehicles).

The qualitative part of the study confirmed findings from the survey regarding motivators for cycling. In particular, “occasional” cyclists attached a great deal of value to the fact that bicycles allowed them to save time and avoid traffic jams: “This saves me time and stress too (…), because I just hop on my bike and don’t even think about buses that are stuck in traffic and the people that barely fit inside them”. They also perceived cycling as a cheaper and more comfortable way of moving in the city, allowing to reach places difficult to access by other means of transport and to avoid parking and fuel costs. These practical aspects of cycling were complemented by less tangible, but still very important, benefits of cycling, such as the feeling of independence and freedom. Moving around the city by bicycle simply made the interviewees feel good.

Additionally, “hard core” users valued predictability and reliability of cycling, no matter what happened on the road. As explained by one of the interviewees: “(…) transportation time on a bicycle is very predictable. If the city is paralysed by snow, I ride for 25 min. If there is great weather, I ride for 25 min. If there is an awful traffic jam, I ride 25 min. If the pope is visiting, I ride 25 min. This doesn’t happen with any other method of transportation”. Interviewees representing this group, in agreement with cyclists responding to the survey, also underlined the health benefits of cycling as a regular form of physical activity. One “hard core” cyclist also identified the possibility to discover the city from a new perspective as a motivator for cycling: “Especially in the beginning when I switched to biking, I discovered many areas of the city that I never would have reached if I had been riding the tram from point A to B. Even the fact that it is difficult to ride a bike in Warsaw [helps me discover the city], (…) I always look for alternative routes. Maybe here a shortcut through the park, maybe riding on side roads. And you discover new places – a completely different Warsaw. Completely different neighbourhoods, parks, awesome little places”.

The qualitative interviews confirmed survey results on weather conditions as a frequent barrier to cycling for most respondents. In particular, “occasional” cyclists did not use their bike during the winter as it was too cold, unpleasant and dangerous due to slippery surfaces. The rain was also seen as a problem, in particular with regard to wearing appropriate clothing in professional situations at work. Similarly, physical consequences of cycling, such as being sweaty and tired for work were also seen as a problem and thus prevented the use of bikes for utilitarian purposes. Most “hard core” cyclists, however, cycled in Warsaw year-round and did not see weather as a barrier.

Inadequate cycling infrastructure as a barrier to cycling was extensively discussed by the qualitative study participants, which is in line with the findings of the survey. Particularly, lack of bike paths and lanes was seen as a very important barrier to cycling for “occasional” cyclists, while it was not perceived as particularly hindering for “hard core” cyclists who were used to cycling in traffic. It seems that the lack of paths and lanes prevents “occasional” cyclist from active transportation within the city. In general, they believed that most of the bike paths and lanes in Warsaw were built with recreation in mind and not transportation. All interviewees characterised the lanes as too few, badly connected, terminating at unexpected places, and dangerous for those who do not want to cycle among cars in traffic. Interviewees attributed this poor quality of the bike lane network to a lack of a planned and coordinated approach in developing the bicycle-lane network in the city, putting the blame on city officials. Activists claimed that bicycle-lanes were generally built in some spots in the city without a proper consultation. They complained about the lack of a broad coordinated approach to cycle infrastructure planning and mentioned the significant lack of cycling infrastructure within the city centre where it is most dangerous to cycle because of car density.

Inadequate planning for bicycle racks, parking areas and public rentals administered by the city was also mentioned during interviews, with complaints both about insufficient numbers and inadequate locations (mainly found next to supermarkets, and occasionally in the centre or by office blocks). Interviewees suggested that racks should be located, for example, by health clinics, smaller shops, museums or cinemas. In addition, they highlighted the need for supervised bicycle parking areas within office buildings and along the metro line (with a “bike and ride” equivalent to a “park and ride” scheme). While both groups of the interviewees complained about the low numbers of bike racks in the city, “occasional” cyclists were particularly concerned about protecting locked bicycles from theft, which “hard core” cyclist did not seem to worry about.

Moreover, interviewees felt that a change in road regulations to strengthen the rights of cyclists in traffic were needed. They explained that “it could be like it is in Holland, where the responsibility for upholding road safety is mostly placed upon the drivers, (…) what is missing for example, is [the legalization of] a two-part left turn for cyclists to avoid oncoming traffic, which is a case which we weren’t able to win at the Ministry”. Apart from discussing the legislative challenges, the interviewees had also suggestions on how to plan and design the cycle lanes to smoothly incorporate them in the general city’s transport infrastructure, as “the city should invite people [to cycle], through its infrastructure”. These ideas were taken from different European countries and included separation of the cycle lanes from the boundaries of the road, designing lanes enabling two-way movement for bikes, or separating cycle lanes from pavements through the application of a distinctive surface.

Although many respondents were not fully satisfied with the current quality and planning of cycle lanes, they had also noticed a number of positive changes. For example, they appreciated the newly appearing cycle lanes, and the direction in which changes were heading in Warsaw. These improvements were perceived, however, as being asymmetrical, occurring in only some areas of the city. Some interviewees also perceived that the owners of a number of office buildings were promoting, enticing and enabling workers to travel to work by bicycle, thus placing eco-friendly spaces on the map of Warsaw. Interviewees also felt that attitudes towards cycling in Warsaw were becoming increasingly positive, and even seen as modern and “trendy”.

Despite perceiving positive trends, interviewees were highly critical of Warsaw’s driving culture leading to unsafe conditions for cycling. Drivers were perceived as posing regular threats to cyclists on the road: lacking respect and behaving rudely towards them, blocking their lanes, overtaking them at a distance of only a few centimetres, and not paying attention to their needs (such as feeling safe). The driving culture in Warsaw can be generally called, as one interviewee expressed that: larger and faster rule on the streets”. Another interviewee framed it in a following way: “I am under the impression that when I ride the road, everyone, that is to say drivers, are very unfriendly because [the road] is their place and I am bothering them in some way” and underlined that “this is simply a city [made] for cars”. Pedestrians were also accused of not paying attention to cyclists and often using bike paths without any consideration for their actual purpose. As a result, many of the “occasional” cyclists feeling unsafe when cycling on the roads ended up frequently riding on sidewalks, thus in turn contributing to unfriendly pedestrian attitudes towards cyclists.

Although “hard core” cyclists also believed that “Traffic on the streets [of Warsaw] is pretty wild”, they seemed to feel less threatened by the traffic due to their larger experience, as they cycled more frequently and longer distances than the “occasional” cyclists. They felt their experience as cyclists enabled them to learn how to accurately predict possible hazards posed by the drivers. They indicated, however, that riding on the streets required constant awareness and concentration, and felt that this inability to focus on anything other than safety while cycling was a disadvantage in comparison with public transportation. Hence, both groups of cyclists coincided in the need for drivers to expand their awareness of cycling safety issues and increase their willingness to share the roads.

In general, Warsaw was not perceived as a very cycling-friendly city. It was described as very spread-out with fairly low concentration of inhabitants and thus average travel to work or school took relatively long time. Compared to other large Polish cities, such as Cracow, Poznan or Wroclaw, Warsaw was perceived as least adapted to cycling.

## 4 Discussion

### 4.1 Cyclist types and characteristics

We surveyed a random population sample and a sample of cyclists, followed by in-depth qualitative interviews of cyclists and cycling experts in Warsaw, Poland, with the aim to better understand characteristics and perceptions of different groups of cyclists and non-cyclists in a large, CEE city. Existing data from other large cities in CEE countries show similar, low levels of cycling (Appendix, Table S1). For example, in Budapest, Hungary, modal share of cycling was 1% (Puhe and Schippl, 2014), although it has been increasing rapidly recently (Juhász et al., 2014). Similarly, in Sofia, Bulgaria, and in Prague, the Czech Republic, the share of cycling was ca. 1% of all trips (Barnfield and Plyushteva, 2016, Vojtěch and Susilo, 2016). Nevertheless, there are few studies investigating cycling in Eastern Europe, so data on cycling rates are rarely available.

We compared cyclists to non-cyclists in our random sample, and recreational to utilitarian cyclists in the cyclist sample, as well as identified two contrasting types of cyclists – “occasional” and “hard core” in the qualitative study. There can be different ways of segmenting cyclists, depending on the purpose of the segmentation (Christmas et al., 2010). Most commonly different groups are distinguished based on their behaviour, motivation and characteristics (Gatersleben and Haddad, 2010), with particular focus on intensity of bicycle use (Damant-Sirois et al., 2014). In addition to the common cyclist versus non-cyclist segmentation, we were particularly interested in our quantitative survey of cyclists in contrasting utilitarian and recreational cyclists. This choice was based on an interest in understanding how to promote cycling for transport as a seamless way to integrate physical activity into a daily routine (Gerike et al., 2016), and in response to cycling behaviours and conditions in Warsaw being particularly geared towards recreational cycling. On the other hand, the qualitative study segmentation was not pre-set but emerged from the analysis of specific context in Warsaw where there is increasing number of people who cycle occasionally and a few who cycle all year round. Our segmentations mirror in part groupings proposed by Winters et al. (2011), who called everyone who had not ridden a bicycle in the past year as a “potential cyclist,” while all others were: occasional, frequent or regular, and by Heinen et al. (2011) who use three groups: non-cyclists, full-time cyclists (every working day), and part-time cyclists (at least once a year).

We found that a large proportion of Warsaw residents cycled at least occasionally, but that cycling was mostly used for recreational purposes, which is typical of regions with very low rates of cycling, such as the USA (e.g. Pucher et al., 1999, Moudon et al., 2005), while as in European cities cycling is most commonly seen as a mode of travel (Rietveld and Daniel, 2004). Respondents in Warsaw did, however, feel that efforts were being made to increase commuter cycling in the city, similar to trends observed in Western Europe (Pucher and Buehler, 2008, Clark et al., 2016, Yang et al., 2017), but also in the USA (Xing et al., 2010, Manaugh et al., 2017, Hirsch et al., 2017).

Most cyclists were female (Table 1) in our random sample, but this is predominantly due to larger female proportion of recreational cyclists. As in other cities where cycling is a marginal means of transportation, utilitarian cycling in Warsaw was male-dominated, although to a much lesser extent than in other such cities (Pucher et al., 2011). This presents quite a unique opportunity for cycle promotion in Warsaw, as convincing women to cycle is often seen as an important step in achieving healthy cycling rates in cities (Aldred et al., 2017).

Utilitarian cycling appeared to take place in addition to recreational forms of activity (no difference in vigorous activity across groups), which supports nascent literature showing utilitarian cycling does not substitute other forms of activity (Donaire-Gonzalez et al., 2015). Healthier weight status of cyclists vs non-cyclists and utilitarian cyclists vs recreational cyclists also corroborates recent literature finding (Flint et al., 2016).

### 4.2 Perceptions of cycling

In line with previous findings, we found that cyclists were significantly more likely to state that friends and family would cycle with them, indicating that social support and positive social norm around cycling can play a key role in encouraging cycling in Warsaw (Heinen et al., 2010; Goetschi et al., 2017). The second strongest motivator for cycling was the perception of cycling as a means to promote health, which was also confirmed in qualitative interviews. This is in accordance with a limited number of previous studies (e.g. Heinen et al., 2010; Heinen et al., 2011). We also found cyclists to be more likely to believe cycling would contribute to reducing air pollution, which indicates altruistic beliefs about cycling may be a motivating factor to cycle in Warsaw. This is supported by existing literature on altruism and use of public transport, with indications of relevance for non-motorized travel as well (Heinen et al., 2010, Handy et al., 2010, Heinen et al., 2011). Other strong motivators recognized by cyclists in Warsaw and in accordance with previous studies were the rapidity and flexibility associated with cycling (Heinen et al., 2010, Heinen et al., 2011, Handy et al., 2010).

Fear of injury was by far the strongest deterrent to cycling in our sample, in accordance with much of the cycling literature (Heinen et al., 2010). Qualitative interviews further explained the dangerous conditions, in particular a car-oriented culture with little respect for the rights of cyclists. Another common barrier to cycling was the weather. However while rain is usually the most negatively perceived weather phenomena affecting cyclists (Heinen et al., 2011), the qualitative interviews pointed towards cold and icy winter conditions and lack of maintenance of bike lanes, such as removing of snow, as being bigger barrier in Warsaw, deterring even many of the most avid cyclists.

Specific analyses comparing utilitarian and recreational cycling are not common (Handy et al., 2010). The context of a city such as Warsaw, where cycling is perceived mainly as a recreational activity, provided a unique opportunity to further explore how cycling could be promoted for routine transport use. Unlike our comparison between cyclists and non-cyclists, we found few significant differences between the two forms of cycling. The transportation advantages of cycling – i.e. rapidity and flexibility – were the only two statistically significant motivators for utilitarian cycling, highlighting a need for better communications on such advantages for cycle promotion in Warsaw. The qualitative analyses also strongly highlighted the importance of flexibility and rapidity afforded by cycling to go places – along with other benefits not captured in typical surveys such as the joy of discovering the city on a bicycle. There are clear opportunities to promote cycling in the city by making such benefits more salient.

Weather conditionswas the only significant deterrent for utilitarian versus recreational cycling, with, again, the particular harsh winter conditions further highlighted in the qualitative interviews. An effort to remove snow from bike facilities would clearly be needed for cycling to be perceived and used as a daily transport mode in Warsaw.

In general, people who cycled at least occasionally tended to perceive more benefits and fewer barriers to cycling than non-cyclists, and cycling for utilitarian purposes enhanced further these beliefs. As expected, differences between recreational and utilitarian cyclists were much less pronounced than between cyclists and non-cyclists.

### 4.3 Cycling infrastructure

Well-connected bike lanes separated from traffic and proper integration of cycling with public transport are indeed the most common features associated with higher levels of cycling (Cervero and Duncan, 2003, Damant-Sirois et al., 2014, Fishman et al., 2012, Gatersleben and Haddad, 2010 Horton, 2007, Rietveld and Daniel, 2004, Pucher et al., 2010, Margués et al., 2015, Manaugh et al., 2017). Good cycling infrastructure helps in particular to promote cycling by providing a sense of safety. Cities that have implemented infrastructure policies aiming at promotion of citywide cycling have reported significant safety benefits with increased cycling volumes over time (Viola et al., 2010, WRI, 2013). This may be due to infrastructure offering a protected space for cycling, in addition to potentially increasing cycle awareness, normalizing the behaviour, and increasing numbers of cyclists which in turn provides “safety in numbers” (Elvik and Bjørnskau, 2017). Perhaps surprisingly, we found no significant relationship between cyclist status (i.e. cyclist versus non-cyclist and utilitarian versus recreational cyclist) and perception of adequate cycle lane infrastructure in our quantitative survey. The qualitative interviews, however, led to ample discussion on the safety problems associated with cycling in the city. Perhaps the dominant perception of cycling as a recreational activity led cyclists to disregard the lack of bike lanes as a deterrent to cycling in our sample. The lack of shower facilities at work, which has been shown to be important in other studies (Buehler, 2012), was similarly not a significantly deterrent in our sample. Availability of bike parking facilities, on the other hand, led to greater odds of being a cyclist, in accordance with the literature (Buehler, 2012, Braun et al., 2016, Heinen et al., 2010).

Lack of proper infrastructure for cyclists in Warsaw resembles other large cities of CEE: in Budapest, Hungary, and in Sofia, Bulgaria, cyclists underlined the need for improvements in the cycling infrastructure as a factor potentially encouraging more cycling within the city (Puhe and Schippl, 2014, Barnfield and Plyushteva, 2016). Similarly, infrastructure was one of the most important issues highlighted in the study of cycling in another large Polish city, Cracow, where most people who cycle do it for utilitarian reasons (Małochleb et al., 2013).

### 4.4 Promoting cycling in the city: for people and with people

Our analyses exposed important areas of future policy development to help promote cycling in Warsaw. The results show that to encourage more people to cycle in Warsaw, a holistic approach with a variety of activities is needed, ranging from improvements in cycling infrastructure (including winter maintenance), changes in driving culture, and promotional campaigns making benefits of utilitarian cycling more salient. This is in line with the previous literature (Pucher et al., 1999, Pucher et al., 2010, WRI, 2013), showing that policy “packages” with multiple approaches from infrastructure to targeted campaigns produce best results.

Rietveld and Daniel (2004) in addition suggest that a combination of “push and pull” policies is necessary to encourage cycling, i.e. not only the improvements of proper infrastructure, but also making other modes of transport more costly and less comfortable, by, for example, having expensive parking areas, making them more scarce or introducing car speed limits. The other potential way in which cycling has become more popular in low-cycling settings is through the introduction of bicycle sharing systems – increasingly popular around the world (Shaheen et al., 2012, Fishman, 2016) public bike system allows short-term bicycle rental between docking stations, and so make cycling more normalized in as a form of public transport. This is also the case of Warsaw, where the Veturilo public bike system opened in 2012 and registered 2.5 million rentals until April 2014 and number of users is growing: as much as 2.23 million rentals were recorded from March to June 2017 (more than in 2016 year altogether) (https://www.veturilo.waw.pl/10-milionow-wypozyczen-veturilo/ accessed 28.06.2017). The bicycle sharing system was opened after the field work of the present study was done and there is a need for future research about its impact on cycling in Warsaw.

Our analyses particularly highlighted the importance of the cultural environment related to cycling. The survey identified social support (as measured by friends and family cycling) as the largest motivating factor distinguishing cyclists from non-cyclists, which is in line with the literature on the importance of social support as a determinant of cycling (Winters et al., 2017, Willis et al., 2015). Qualitative analyses revealed a problematic lack of respect of both drivers and pedestrians towards cyclists, making cyclists feel unsafe, and not welcomed within the city. These findings indicate that legislation and infrastructural changes may not be enough to improve cycling rates in Warsaw. What is also needed is a change in people’s thinking, improving public acceptability of cycling and changing public norms, for example through relevant information and education campaigns (Aldred and Jungnickel, 2014, Iwińska and Troszyński, 2014, Daley and Rissel, 2011.

However, information and education, as one-way processes, are not sufficient determinants of the behavioural change of the drivers. Change in behaviour is not an “event”, but a longer-term process characterised by different stages, from the pre-contemplation stage where the subject of change is not aware of the problem and has no intention to change, through contemplation, preparation to change and action, to maintenance of the desired behaviour (Daley and Rissel, 2011; Prochaska et al., 1994; Holladay, 2002). To direct a process of change a comprehensive set of strategies different for particular stages needs to be applied, such as increase in awareness, motivating, development of specific plans of action, feedbacks and social support (Gatersleben and Appleton, 2007). Thus, although education and information are necessary to increase awareness at the beginning of the process of change, other strategies are also indispensable.

## 5 Conclusions

The results of the study can be summarized with four key recommendations, relevant to other cities, in particular in Eastern Europe, where cycling is mostly a recreational activity and rates are relatively low.

First, communication around cycling is likely to be most resonant in the population of Warsaw when focused on the promotion of cycling as a healthy and sustainable lifestyle. This could be the cornerstone towards changing mindsets towards sustainable and active transportation and include an effort to change drivers’ attitudes towards cyclists.

Second, such communication efforts should include an emphasis on benefits of cycling as a rapid and convenient mean of transportation, presenting cycling as a travel mode and not solely as a recreational activity.

Third, the development of improved cycling infrastructure considering the needs of cyclists incorporated in a holistic city planning is necessary and emphasis should be given above all to the safety issues, connectivity of paths and lanes, and maintenance of the infrastructure, including in the winter.

Finally, future planning efforts should integrate knowledge and perception from users and potential users, and not solely be developed by experts and technicians.

## Footnotes

1 TomTom Congestion Index measures extra travel time between congested and uncongested conditions in 390 cities worldwide.

2 According to Warsaw municipality website: http://transport.um.warszawa.pl/standardy-rowerowe/ruch-rowerowy (accessed 18.12.2017).

3 According to research ethical guidance, we guaranteed promises of confidentiality and informed consent of the participants; all interviews are presented here after a procedure of data anonymization.
