# Supplementary material for: Cycling in Warsaw, Poland – Perceived enablers and barriers according to cyclists and non-cyclists
Source: Transp Res Part A Policy Pract. 2018 Jul;113:291–301. doi: 10.1016/j.tra.2018.04.014 (PMC6039858; doi:10.1016/j.tra.2018.04.014)
Supplement: Supplementary data 1 [file mmc1.docx]

**Table S1**: Use of different modes of transport (in %) in the selected Central Eastern Europe (CEE) cities (source: EPOMM Modal Split Tool from <http://www.epomm.eu/tems/compare_cities.phtml> ) data accessed 11/01/2018.

| **City** | **Country** | **Year** | **Population** | **Walk (%)** | **Bike (%)** | **Public**  **Transport (%)** | **Car (%)** |
| --- | --- | --- | --- | --- | --- | --- | --- |
| Sofia | Bulgaria | 2010 | 1.211.348 | 14 | 3 | 32 | 51 |
| Belgrade | Serbia | 2015 | 1.659.440 | 24 | 1 | 49 | 26 |
| Bucharest | Romania | 2007 | 1.940.000 | 22 | 1 | 53 | 24 |
| Budapest | Hungary | 2014 | 1.744.655 | 18 | 2 | 45 | 35 |
| Warsaw | Poland | 2005 | 1.702.000 | 21 | 1 | 54 | 24 |

**Table S2:** Survey variable definitions

| Variable | **Random Sample** | | **Cyclist Sample** | |
| --- | --- | --- | --- | --- |
| ***Cyclist categories*** | Non-cyclist | Did not cycle in the past 6 months (neither recreational nor to go to places). | Recreational | Cycled at least once in the past 6 months for recreational purposes only. |
|  | Cyclist | Cycled at least once in the past 6 months to go to places, recreational or both. | Utilitarian | Cycled at least once in the past 6 months either to go to places (utilitarian travel) or both to go to places and for recreational purposes). |
| ***Total duration in minute cycling per week*** | NA as the variables needed were poorly recoded. | | Those who have ridden a bike in the last 7 days before responding the survey | Number of days cycle per week (0-7) * Average amount of time per day cycling (minutes) |
|  |  |  | Those who have not ridden a bike in the last 7 days before responding the survey | Average amount of time per day cycling in the past 6 months / 4.33 (average number of weeks in a month). |
| ***Total duration in minute per week of Vigorous physical activity*** | number of days in a week* total minute of vigorous physical activity per day | | | |
| ***Total duration in minute per week of Moderate physical activity*** | number of days in a week * total minute of moderate physical activity per day | | | |
| ***BMI categories in (BMI (kg/m^2))*** | 14 to 18.5 (kg/m^2)  18.5 to 25(kg/m^2)  25 to 50 (kg/m^2) | | Underweight  Normal  Overweight | |

**Table S3**: Perceptions of cycling across cyclists and non-cylists (random survey) and across recreational and utilitarian cyclist (cyclist survey).

|  | Random sample | | | | Cyclist sample | | | |
| --- | --- | --- | --- | --- | --- | --- | --- | --- |
|  | N(%) | Non-cyclist | Cyclist | p-value | N(%) | Recreational | Utilitarian | p-value |
| I would never consider riding a bicycle in Warsaw |  |  |  |  |  |  |  |  |
| Disagree | 307(57%) | 65(28%) | 241 (80%) |  | 445(90%) | 180(88%) | 265(90%) |  |
| Agree | 227(42%) | 167(72%) | 59 (20%) | P<0.001 | 52(10%) | 24(12%) | 28(10%) | P=0.521 |
| Bicycling will improve my physical and mental condition |  |  |  |  |  |  |  |  |
| Disagree | 54(10%) | 45(20%) | 7(2%) |  | 16(3%) | 7(3%) | 9(3%) |  |
| Agree | 472(90%) | 176(80%) | 296 (98%) | P<0.001 | 485(97%) | 197(97%) | 288(97%) | P=1 |
| I have friends and/or family members who will bicycle with me |  |  |  |  |  |  |  |  |
| Disagree | 120(22%) | 100(43%) | 19 (6%) |  | 60(12%) | 27(13%) | 33(11%) |  |
| Agree | 418(78%) | 134(57%) | 283(94%) | P<0.001 | 429(88%) | 174(87%) | 255(89%) | P=0.607 |
| Weather conditions frequently prevent me from bicycling. |  |  |  |  |  |  |  |  |
| Disagree | 112(21%) | 41(17%) | 70(23%) |  | 178(37%) | 60(30%) | 118(41%) |  |
| Agree | 430(79%) | 199(83%) | 230(77%) | P=0.086 | 306(63%) | 139(70%) | 167(59%) | P=0.015 |
| The risk of injury prevents me from bicycling. |  |  |  |  |  |  |  |  |
| Disagree | 246(45%) | 62(26%) | 183(61%) |  | 432(87%) | 177(88%) | 255(86%) |  |
| Agree | 299(55%) | 180(74%) | 118(39%) | P<0.001 | 65(13%) | 25(12%) | 40(14%) | P=0.803 |
| I will spend less money on transportation if I travel by bicycle |  |  |  |  |  |  |  |  |
| Disagree | 158(30%) | 101(44%) | 57(19%) |  | 70(14%) | 34(17%) | 36(12%) |  |
| Agree | 374(70%) | 131(56%) | 241(81%) | P<0.001 | 421(86%) | 164(82%) | 257(88%) | P=0.165 |
| I do not have the time or resources to maintain my own bicycle |  |  |  |  |  |  |  |  |
| Disagree | 256(48%) | 70(30%) | 186(62%) |  | 420(86%) | 174(87%) | 246(84%) |  |
| Agree | 283(53%) | 166(70%) | 115(38%) | P<0.001 | 71(14%) | 25(13%) | 46(16%) | P=0.392 |
| Bicycling will improve the air quality in my immediate area |  |  |  |  |  |  |  |  |
| Disagree | 56(11%) | 37(16%) | 18(6%) |  | 73(15%) | 31(16%) | 42(15%) |  |
| Agree | 475(89%) | 193(84%) | 282(94%) | P<0.001 | 407(85%) | 167(84%) | 240(85%) | P=0.923 |
| The number of pedestrians on the sidewalks makes it difficult for me to commute to my regular destinations. |  |  |  |  |  |  |  |  |
| Disagree | 90(17%) | 54(23%) | 36(12%) |  | 186(39%) | 77(41.18%) | 109(38%) |  |
| Agree | 447(83%) | 183(77%) | 263(88%) | P=0.001 | 290(61%) | 110(58.82%) | 180(62%) | P=0.509 |
| The combination of the bicycle with another mode of transport will gain me greater flexibility and freedom in everyday transportation |  |  |  |  |  |  |  |  |
| Disagree | 110(21%) | 78(34%) | 31(10%) |  | 98(21%) | 57(30%) | 41(14%) |  |
| Agree | 418(79%) | 152(66%) | 266(90%) | P<0.001 | 377(79%) | 133(70%) | 244(86%) | P<0.001 |
| The bicycle allows me to reach destinations more quickly than by using other travel modes. |  |  |  |  |  |  |  |  |
| Disagree | 137(25%) | 90(38%) | 45(15%) |  | 173(36%) | 95(50.%) | 78(27%) |  |
| Agree | 402(75%) | 147(62%) | 255(85%) | P<0.001 | 302(64%) | 94(50%) | 208(73%) | P<0.001 |
| I have adequate bicycle parking and storage facilities at the place where I live. |  |  |  |  |  |  |  |  |
| Disagree | 183(34%) | 110(47%) | 71(24%) |  | 155(31%) | 73(36%) | 82(28%) |  |
| Agree | 356(66%) | 125(53%) | 231(76%) | P<0.001 | 345(69%) | 132(64%) | 213(72%) | P=0.078 |
| A lack of shower facilities at destinations keeps me from bicycling. |  |  |  |  |  |  |  |  |
| Disagree | 189(37%) | 84(39%) | 103(35%) |  | 275(57%) | 108(55%) | 167(58%) |  |
| Agree | 321(63$) | 134(61%) | 187(64%) | P=0.546 | 211(43%) | 89(45%) | 122(42%) | P=0.579 |
| The difficulty of maintaining personal appearance while riding a bicycle often keeps me from bicycling. |  |  |  |  |  |  |  |  |
| Disagree | 143(26%) | 57(24%) | 85(28%) |  | 311(63%) | 119(59%) | 192(66%) |  |
| Agree | 399(74%) | 183(76%) | 215(72%) | P=0.297 | 184(37%) | 84(41%) | 100(34%) | P=0.128 |
| The lack of bicycle lanes keeps me from bicycling. |  |  |  |  |  |  |  |  |
| Disagree | 77(14%) | 44(18%) | 33(11%) |  | 202(40%) | 83(40%) | 119(40%) |  |
| Agree | 465(86%) | 197(82%) | 267(89%) | P=0.023 | 297(60%) | 122(60%) | 175(60%) | P=1 |

**Table** **S4:**  Multivariable logistic regressions of association for cyclist vs non-cyclist and individual characteristics in the random sample.

|  | Models adjusted for age and sex | | | Models adjusted for age, sex and BMI* | | |
| --- | --- | --- | --- | --- | --- | --- |
|  | OR | 95% CI | p-value | OR | 95% CI | p-value |
| I would never consider riding a bicycle in Warsaw  agree | 0.11 | (0.07; 0.17) | P<0.001 | 0.10 | (0.07; 0.16) | P<0.001 |
| Bicycling will improve my physical and mental condition  agree | 9.96 | (4.55; 25.12) | P<0.001 | 8.23 | (3.66; 21.18) | P<0.001 |
| I have friends and/or family members who will bicycle with me  agree | 10.66 | (6.27; 18.99) | P<0.001 | 10.18 | (5.89; 18.40) | P<0.001 |
| Weather conditions frequently prevent me from bicycling.  agree | 0.71 | (0.44; 1.11) | P=0.132 | 0.46 | (0.27; 0.79) | P=0.005 |
| The risk of injury prevents me from bicycling.  agree | 0.25 | (0.17; 0.37) | P<0.001 | 0.21 | (0.13; 0.32) | P<0.001 |
| I will spend less money on transportation if I travel by bicycle  agree | 3.55 | (2.36; 5.39) | P<0.001 | 3.42 | (2.22; 5.32) | P<0.001 |
| I do not have the time or resources to maintain my own bicycle  agree | 0.29 | (0.20; 0.42) | P<0.001 | 0.28 | (0.18; 0.42) | P<0.001 |
| Bicycling will improve the air quality in my immediate area  agree | 3.14 | (1.70; 5.98) | P<0.001 | 2.88 | (1.48; 5.74) | P=0.002 |
| The number of pedestrians on the sidewalks makes it difficult for me to commute to my regular destinations.  agree | 2.44 | (1.50; 4.01) | P<0.001 | 2.21 | (1.25; 3.95) | P=0.007 |
| The combination of the bicycle with another mode of transport will gain me greater flexibility and freedom in everyday transportation  agree | 4.15 | (2.59; 6.79) | P<0.001 | 3.90 | (2.33; 6.65) | P<0.001 |
| The bicycle allows me to reach destinations more quickly than by using other travel modes.  agree | 3.45 | (2.25; 5.36) | P<0.001 | 3.39 | (2.13; 5.47) | P<0.001 |
| I have adequate bicycle parking and storage facilities at the place where I live.  agree | 2.63 | (1.79; 3.88) | P<0.001 | 2.79 | (1.84; 4.26) | P<0.001 |
| A lack of shower facilities at destinations keeps me from bicycling.  agree | 1.14 | (0.78; 1.68) | P=0.472 | 1.18 | (0.77; 1.77) | P=0.469 |
| The difficulty of maintaining personal appearance while riding a bicycle often keeps me from bicycling.  agree | 0.73 | (0.48; 1.10) | P=0.137 | 0.65 | (0.40; 1.03) | P=0.069 |
| The lack of bicycle lanes keeps me from bicycling.  agree | 1.15 | (0.78; 1.68) | P=0.472 | 1.37 | (0.75; 2.50) | P=0.300 |

* BMI adjusted as a continuous variable

**Table S**5: Multivariable logistic regressions of association between utilitarian vs recreational cyclist and individual characteristics in the cyclist sample.

|  | Models adjusted for age and sex | | | Models adjusted for age, sex and BMI categories* | | |
| --- | --- | --- | --- | --- | --- | --- |
|  | OR | 95% CI | p-value | OR | 95% CI | p-value |
| I would never consider riding a bicycle in Warsaw  agree | 0.93 | (0.52; 1.70) | P=0.815 | 0.93 | (0.51; 1.71) | P=0.805 |
| Bicycling will improve my physical and mental condition  agree | 1.23 | (0.43; 3.41 ) | P=0.689 | 0.94 | (0.30; 2.73) | P=0.908 |
| I have friends and/or family members who will bicycle with me  agree | 1.23 | (0.70; 2.14) | P=0.451 | 1.25 | (0.71; 2.19) | P=0.426 |
| Weather conditions frequently prevent me from bicycling.  agree | 0.60 | (0.40; 0.88) | P=0.009 | 0.61 | (0.41; 0.90) | P=0.014 |
| The risk of injury prevents me from bicycling.  agree | 1.25 | (0.73; 2.18) | P=0.427 | 1.36 | (0.78; 2.42) | P=0.284 |
| I will spend less money on transportation if I travel by bicycle  agree | 1.51 | (0.90; 2.53) | P=0.117 | 1.55 | (0.91; 2.62) | P=0.106 |
| I do not have the time or resources to maintain my own bicycle  agree | 1.27 | (0.75; 2.19) | P=0.384 | 1.37 | (0.79; 2.42) | P=0.260 |
| Bicycling will improve the air quality in my immediate area  agree | 1.59 | (0.70; 1.93) | P=0.574 | 1.18 | (0.67; 1.99) | P=0.531 |
| The number of pedestrians on the sidewalks makes it difficult for me to commute to my regular destinations.  agree | 1.23 | (0.84; 1.81) | P=0.288 | 1.28 | (0.86; 1.90) | P=0.227 |
| The combination of the bicycle with another mode of transport will gain me greater flexibility and freedom in everyday transportation  agree | 2.61 | (1.65; 4.17) | P<0.001 | 2.63 | (1.65; 4.23) | P<0.001 |
| The bicycle allows me to reach destinations more quickly than by using other travel modes. | 2.85 | (1.92; 4.25) | P<0.001 | 2.98 | (2.00; 4.49) | P<0.001 |
| I have adequate bicycle parking and storage facilities at the place where I live.  agree | 1.37 | (0.93; 2.03) | P=0.109 | 1.31 | (0.88; 1.95) | P=0.181 |
| A lack of shower facilities at destinations keeps me from bicycling.  agree | 0.86 | (0.60; 1.25) | P=0.437 | 0.87 | (0.59;1.27) | P=0.467 |
| The difficulty of maintaining personal appearance while riding a bicycle often keeps me from bicycling.  agree | 0.75 | (0.51; 1.09) | P=0.31 | 0.75 | (0.51; 1.10) | P=0.142 |
| The lack of bicycle lanes keeps me from bicycling.  agree | 1.07 | (0.73; 1.55) | P=0.73 | 1.11 | (0.75; 1.62) | P=0.602 |

* BMI adjusted as a categorical variable
